# Safety, Tolerability, and Immunogenicity of Booster Dose with MVC-COV1901 or MVC-COV1901-Beta SARS-CoV-2 Vaccine in Adults: A Phase I, Prospective, Randomized, Open-Labeled Study

**DOI:** 10.3390/vaccines11121798

**Published:** 2023-12-01

**Authors:** Chia En Lien, Ming-Che Liu, Ning-Chi Wang, Luke Tzu-Chi Liu, Chung-Chin Wu, Wei-Hsuan Tang, Wei-Cheng Lian, Kuan-Ying A. Huang, Charles Chen

**Affiliations:** 1 Medigen Vaccine Biologics Corporation, Taipei 114, Taiwan; 2 Institute of Public Health, National Yang-Ming Chiao Tung University, Taipei 112, Taiwan; 3 Clinical Research Centre, Taipei Medical University Hospital Taipei, Taipei 110, Taiwan; 4 School of Dental Technology, College of Oral Medicine, Taipei Medical University, Taipei 110, Taiwan; 5 Tri-Service General Hospital, Taipei 114, Taiwan; 6 Graduate Institute of Immunology, College of Medicine, National Taiwan University, Taipei 100, Taiwan; 7 Department of Pediatrics, National Taiwan University Hospital, Taipei 100, Taiwan; 8 College of Science and Technology, Temple University, Philadelphia, PA 19122, USA

**Keywords:** COVID-19, SARS-CoV-2 vaccine, MVC-COV1901, booster vaccination

## Abstract

Severe acute respiratory syndrome coronavirus 2 (SARS-CoV-2) vaccines based on variant strains have been in use as booster doses to update immunity against circulating variants. Here we present the results of a phase one prospective, randomized, and open-labeled trial to study the safety and immunogenicity of a booster dose consisting of a subunit vaccine based on the stabilized prefusion SARS-CoV-2 spike protein, MVC-COV1901, or its Beta version, MVC-COV1901-Beta. Participants aged ≥18 and <55 years who received two or three prior doses of MVC-COV1901 vaccines were enrolled and were to receive a booster dose of either 15 mcg of MVC-COV1901, 15 mcg, or 25 mcg of MVC-COV1901-Beta in a 1:1:1 ratio. Adverse reactions after either MVC-COV1901 or MVC-COV1901-Beta booster doses after two or three doses of MVC-COV1901 were comparable and mostly mild and transient. At four weeks after the booster dose, participants with two prior doses of MVC-COV1901 had higher levels of neutralizing antibodies against ancestral SARS-CoV-2, Beta, and Omicron variants than participants with three prior doses of MVC-COV1901, regardless of the type of booster used. MVC-COV1901 and MVC-COV1901-Beta can both be effectively used as booster doses against SARS-CoV-2, including the BA.4/BA.5 Omicron variants.

## 1. Introduction

In May 2023, the World Health Organization (WHO) declared that COVID-19 is no longer a global health emergency and is instead an established and ongoing health issue [1]. Vaccination has been an effective weapon against this pandemic, and as of November 2023, over 13 billion doses of COVID-19 vaccines have been administered [2]. Even though the world has largely returned to normal, pre-pandemic life, the ever-changing nature of SARS-CoV-2 to escape vaccine-induced antibody neutralization is the driving force behind the diversification of virus variants [3,4]. In late 2023, almost all circulating SARS-CoV-2 variants will be descended from the Omicron lineage, which includes the earlier BA.2 and BA.4/BA.5 variants and the current EG.5, BA.2.86, and recombinant XBB.1.5 and XBB.1.16 variants [5]. Several vaccine manufacturers have rolled out Omicron XBB.1.5-based vaccines to update the immunity against the newer variants, and preliminary data have shown that the XBB.1.5 booster can induce broad neutralizing activity against multiple XBB variants and BA.2.86. [6,7,8]. However, the possibility still exists that previous VOCs such as Beta and Delta variants could re-emerge or form recombinants with Omircon variants capable of causing renewed outbreaks [9]. As the booster doses shifted from the bivalent vaccine to the monovalent XBB vaccine in 2023, instead of engaging in the never-ending quest for pursuing after variants, it is important to look back at previous VOCs as sources of broad spectrum activity as futureproof against emerging variants.

MVC-COV1901 is a subunit COVID-19 vaccine based on the stable prefusion spike protein S-2P of the ancestral SARS-CoV-2 and adjuvanted with CpG 1018 and aluminum hydroxide have previously shown that three doses of MVC-COV1901 can improve the neutralizing antibody response against live SARS-CoV-2 and Omicron variant pseudoviruses [10]. The current study builds on the observation that the Beta variant version of S-2P protected hamsters from the Delta variant challenge and improved neutralizing antibody levels against the Omicron variant [11]. For this study, two groups of participants who have received either two or three doses of MVC-COV1901 were administered a booster dose of either original MVC-COV1901 or MVC-COV1901 based on the Beta variant in two different dose levels (MVC-COV1901-Beta). We carried out this study to investigate the reactogenicity and immunogenicity against the original SARS-CoV-2 and the Beta variant after the booster doses.

## 2. Materials and Methods

### 2.1. Study Design and Participants

This was a prospective, randomized, open-labeled phase I study to evaluate the safety, tolerability, and immunogenicity of a booster dose of the MVC-COV1901 or MVC-COV1901-Beta SARS-CoV-2 vaccine in adult participants. Approximately 120 participants were screened, and participants who received two or three prior doses of MVC-COV1901 were respectively placed into Group A or Group B. Eligible participants were healthy adults or adults with pre-existing medical conditions who were in stable condition and aged from 18 (inclusive) to 55 years. The participants in Group A were those who have received two doses of MVC-COV1901 vaccination with 1st and 2nd doses within 12 weeks, while the participants in Group B were those who have received three doses of MVC-COV1901 vaccination with 1st and 2nd doses within 12 weeks and 2nd and 3rd doses between 12 and 24 weeks. Participants in both groups also had the latest dose at least 84 days before randomization and did not receive any other investigational or approved COVID-19 vaccines. This study was carried out at two sites in Taiwan: Taipei Medical University Hospital (Taipei, Taiwan) and the Tri-Service General Hospital (Taipei, Taiwan). This trial was registered at ClinicalTrials.gov as NCT05216601 on 31 January 2022. 

### 2.2. Randomization and Masking

The randomization of each group was stratified based on site to three treatment arms: 15 mcg of MVC-COV1901, or 15 mcg or 25 mcg of MVC-COV1901-Beta in a 1:1:1 ratio. Blinding was not performed as this was an open-labeled study. A stratified permuted block randomization method was used for the generation of a random allocation sequence. Two blocks were used with sizes of 6 and 3, respectively. Randomization was conducted via a sealed envelope with a randomization number and the intervention assignment. The subject was assigned a randomization number according to the chronological order of prescriptions. The subject would know the treatment group only when the site staff opened the randomization envelope. The biostatistician at the contract research organization (CRO) generated the random allocation sequence, and the investigators enrolled participants and assigned participants to interventions via randomization envelope.

### 2.3. Procedure and Outcomes

The investigative product MVC-COV1901 contained 15 mcg of SARS-CoV-2 S-2P protein adjuvanted with CpG 1018 750 mcg and aluminum hydroxide 375 mcg, while MVC-COV1901-Beta contained either 15 mcg or 25 mcg of SARS-CoV-2 Beta variant (B.1.351) S-2P protein adjuvanted with 750 mcg CpG 1018 and 375 mcg aluminum hydroxide. Booster doses were administered as intramuscular injections of 0.5 mL of the vaccine in the deltoid region of the non-dominant arm.

The primary safety endpoint of this study was the incidence of adverse events (AEs) within 28 days of the booster administration. The primary immunogenicity endpoint was the levels of neutralizing antibody titers at Visit 5 (4 weeks after the booster dose) and anti-spike immunoglobulin G (IgG) antibody titers at Visits 4 (2 weeks after the booster dose) and 5. Safety was assessed by incidences of solicited AEs for up to seven days after each vaccination and unsolicited AEs for up to 28 days after each vaccination. Other AEs, such as serious adverse events (SAEs) and adverse events of special interest (AESI), were recorded within this study period. Immunogenicity was assessed by a neutralizing assay with the ancestral (WT) SARS-CoV-2 and Beta variant and IgG titers in terms of geometric mean titer (GMT) and GMT ratio. Pseudovirus neutralization assays with the Omicron variant (BA.4/BA.5 subvariant) pseudovirus were performed with samples from Visits 2 (baseline) and 5. 

Neutralizing antibody titers against live SARS-CoV-2 virus were performed with ancestral SARS-CoV-2 (hCoV-19/Taiwan/4/2020, GISAID EPI_ISL_411927), Beta variant (B.1.351, hCoV-19/Taiwan/1013), and Omicron variant (BA.1, TCDC#16804) [11]. Anti-SARS-CoV-2 spike immunoglobulin (IgG) levels were measured by enzyme-linked immunosorbent assay (ELISA) using custom-made 96-well plates coated with S-2P antigen [12].

Pseudotyped lentivirus with spike proteins of Wuhan wildtype or Omicron (BA.4/BA.5, both possessing identical spike protein sequences) was used in the pseudovirus neutralization assay conducted as reported previously [11]. The mutations for the Omicron variant (BA.4/BA.5) used in the spike sequence for pseudovirus construction were derived from the WHO source [5].

Frozen peripheral blood mononuclear cells were thawed and used to set up the memory B cell (MBC) assay and the enzyme-linked immunospot (ELISpot) assay as previously described [11,12]. A T cell cytokine assay was performed as described previously using ELISpot assay kits specific for IFN-γ and IL-4, and results were expressed as spot-forming units (SFU) per million PBMC [13].

### 2.4. Statistical Analysis

As this was an exploratory phase 1 clinical study, the sample size was arbitrarily determined and was not derived from a statistical estimation method, and a statistical hypothesis was not used for sample size calculation in this study. All results are presented using descriptive statistics. GMT, GMT ratio, and corresponding CI are calculated using an ANCOVA model with baseline log-titers, BMI (<30 or ≥30 kg/m^2^), comorbidity (yes or no), and sex (male or female) as covariates. The GMT ratio is defined as the geometric mean of the fold increase of post-study intervention titers over the baseline titers. Prism 6.01 (GraphPad) was used for statistical analysis. One-way ANOVA, Kruskal–Wallis, and Fisher’s exact test were used to calculate the significance of demographic characteristics (Table 1). Kruskal–Wallis with corrected Dunn’s multiple comparisons test was used for comparison of means of the non-parametric dataset, while the Mann–Whitney U test was used to compare MBC frequencies at two-time points. Linear regression was used to model the relationship between neutralization titer and IgG MBC frequency.

The following groups were used for this study analysis: The safety set included all randomized participants who received this study intervention, and the Full Analysis Set (FAS) included all randomized participants who received this study intervention, irrespective of their protocol adherence and continued participation in this study. Per protocol set (PPS) included all participants in the FAS who received the planned dose of randomized study intervention and, up until Visit 5, did not have laboratory-confirmed COVID-19 infection, were negative for SARS-CoV-2 anti-nucleocapsid tests, and did not have a major protocol deviation that was judged to impact the critical immunogenicity data.

## 3. Results

**Figure 1:**
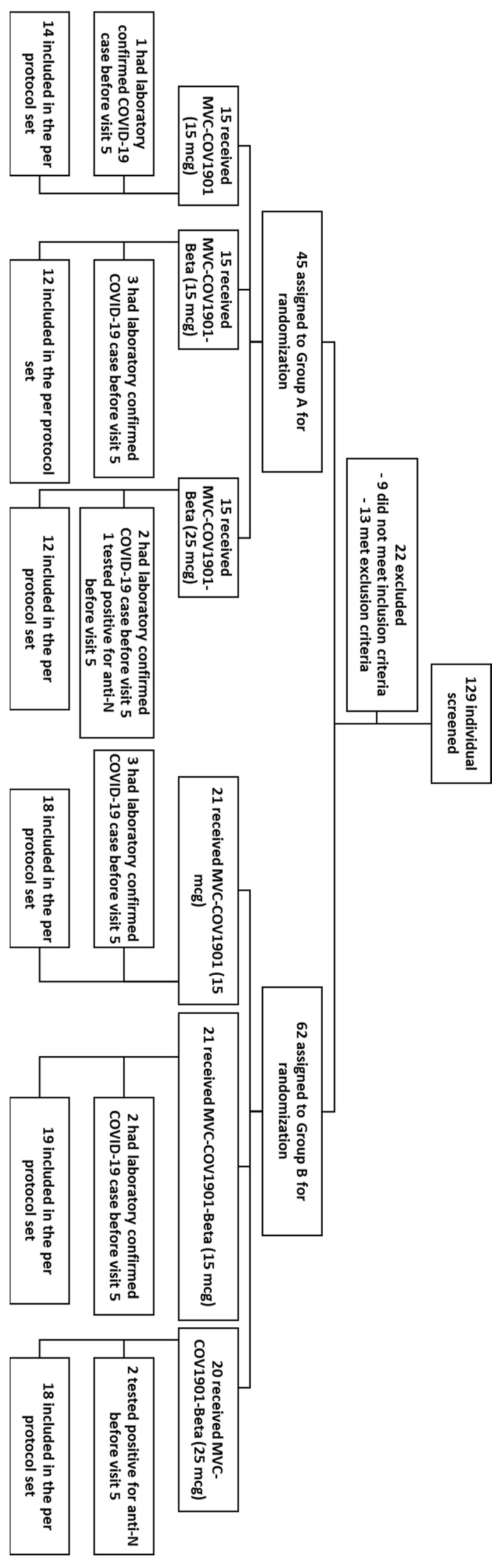
CONSORT flow diagram for this study.

Between May and July 2022, a total of 129 adult participants were screened, and 107 eligible participants were split into groups of 45 and 62 for Groups A and B, respectively (Figure 1). In terms of the demographics of the participants, all groups had similar mean age and BMI levels, although the gender ratios were less equal among the groups (Table 1). The mean intervals between the last dose of MVC-COV1901 and the booster dose were longer in Group A (223.3 to 294.5 days) than in Group B (120.9 to 128.0 days). The differences between the demographic characteristics of the two groups were statistically not significant.

**Figure 2:**
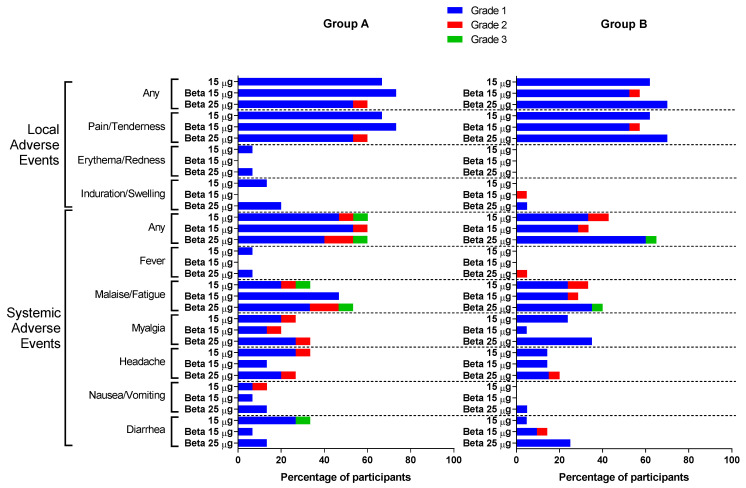
Solicited local and systemic adverse events for each of the treatment groups in Groups A and B.

Solicited adverse events are summarized in Figure 2 and tabulated in Tables S1 and S2, and unsolicited AEs are summarized in Table S3. No SAE (grade 3 AEs or higher) or AESI related to the vaccine have been reported after the booster dose. The most common local and systemic effects after any booster dose were pain/tenderness (60.0~73.3% in Group A and 57.1~70.0% in Group B) and malaise/fatigue (33.3~53.3% in Group A and 28.6~40.0% in Group B), respectively. While erythema/redness (two participants in Group A) and fever (two participants in Group A and one participant in Group B) were the least common AEs, the safety profile and incidences of AEs were comparable in both groups (Figure 2, Tables S1–S3).

**Figure 3:**
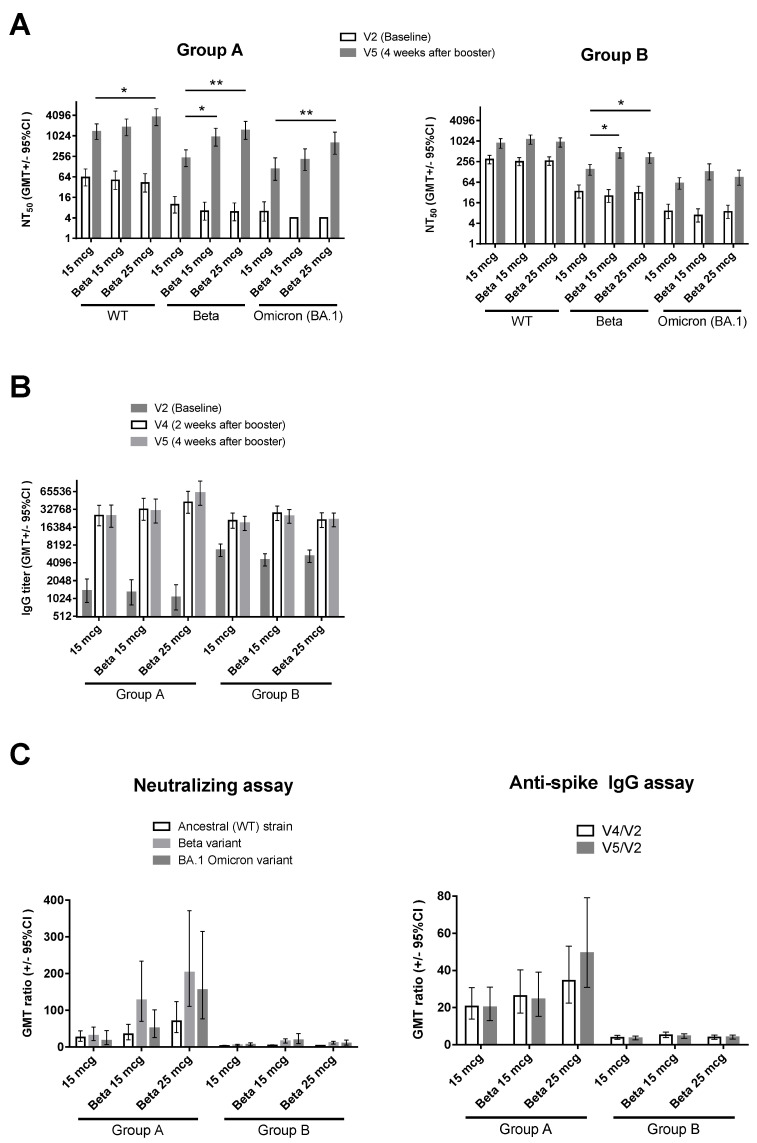
Immunogenicity of the booster dose. (**A**) neutralizing antibody titer against live ancestral (WT) SARS-CoV-2, Beta, and Omicron (BA.1) variants; (**B**) anti-SARS-CoV-2 spike IgG antibody titer; (**C**) GMT ratio of neutralizing antibody titers of V5/V2 (left) and anti-spike IgG titers at V4/V2 and V5/V2 (right). For (**A**,**B**), results are expressed as symbols representing GMT, and error bars represent 95% confidence intervals. For (**C**), results are expressed as the mean GMT ratio with error bars representing 95% confidence intervals. Statistical significance was calculated using the Kruskal–Wallis test with a corrected Dunn’s multiple comparisons test. * = *p* < 0.05, ** = *p* < 0.01.

At V5, Group A participants had numerically higher levels of neutralizing antibodies against the ancestral SARS-CoV-2 (WT), with the GMTs ranging from 1352.0 to 3602.8 for Group A compared to 867.9 to 1125.0 for Group B (Figure 3A and Table S4). Similar results were observed for the Beta variant, with the neutralizing antibody GMTs for Group A ranging from 225.6 to 1476.9 compared to 147.1 to 459.2 in Group B. Neutralization against BA.1 Omicron was also improved, with GMTs in Group A ranging from 116.3 to 609.7 and from 54.9 to 84.9 in Group B (Table S4). In Group A, 15 mcg of MVC-COV1901-Beta resulted in numerically higher levels of neutralizing antibodies against WT and BA.1 live viruses, as well as a significant higher titer against the Beta variant live virus (WT: 1805.0 [95%CI 1023.6–3182.9]; Beta: 931.3 [509.3–1703.0]; BA.1: 190.4 [85.9–421.9]) compared to 15 mcg of MVC-COV1901 (WT: 1352.0 [979.4–2292.4]; Beta: 225.6 [128.1–397.2]; BA.1: 116.3 [55.4–244.0]) (Figure 3A and Table S4). At an increased dose of 25 mcg of MVC-COV1901-Beta, the level of neutralizing antibodies against all of the live viruses tested was significantly increased (WT: 3602.8 [95%CI 2036.7–6373.1]; Beta: 1476.9 [806.4–2704.8]; BA.1: 609.7 [280.7–1324.1]) compared to 15 mcg of MVC-COV1901 (WT: 1352.0 [979.4–2292.4]; Beta: 225.6 [128.1–397.2]; BA.1: 116.3 [55.4–244.0]) (Figure 3A and Table S4). However, in Group B, 15 mcg of MVC-COV1901-Beta induced the highest level of neutralizing antibodies against both Beta and BA.1 variants (Beta: 459.2 [95% CI 322.2–654.6]; BA.1: 124.9 [69.2–225.3]) compared to 15 mcg of MVC-COV1901 (Beta: 147.1 [102.3–211.6]; BA.1: 54.9 [36.5–82.6]) and 25 mcg of MVC-COV1901-Beta (Beta: 323.8 [227.7–460.4]; BA.1: 84.9 [49.5–145.6]) (Figure 3A and Table S4). All participants had high levels of anti-spike IgG at Visits 4 and 5, regardless of the type of booster received or the number of prior doses of MVC-COV1901 (Figure 3B). When calculating the GMT ratio of neutralizing antibodies and IgG titers at V5 or V4 against the baseline (V2) titers, Group B had a minimal increase in GMT ratio compared to Group A (Figure 3C and Table S4). The increase of GMT ratio against the Beta variant was most noticeable in the 25 mcg MVC-COV1901-Beta dosage group for Group A, with V5/V2 neutralizing antibody GMT ratio of 202.9 [110.8–371.5] and V5/V2 IgG GMT ratio of 49.4 [30.9–79.1] in the 25 mcg MVC-COV1901-Beta dosage group compared to V5/V2 neutralizing antibody GMT ratio of 31.0 [17.6–54.6] and V5/V2 IgG GMT ratio of 20.2 [13.1–31.1] in the 15 mcg MVC-COV1901 dosage group (Figure 3C and Table S4). Similar results were noted for the BA.1 variant, in which 25 mcg of MVC-COV1901-Beta resulted in the highest GMT ratio for Group A (152.5 [73.4–316.4] vs. 18.9 [7.4–48.3]) and 15 mcg of MVC-COV1901-Beta for Group B (18.5 [9.1–37.6] vs. 6.2 [3.3–11.3]) when compared to 15 mcg of MVC-COV1901.

**Figure 4:**
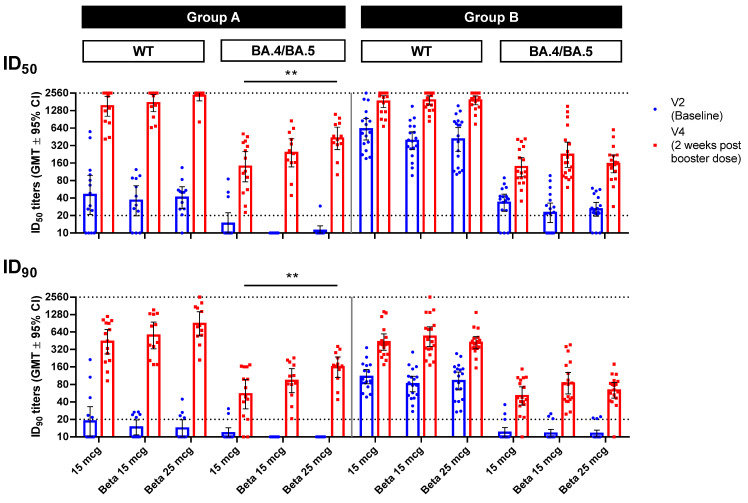
Pseudovirus neutralization assay of pseudovirus with spike proteins of the original SARS-CoV-2 (WT) or Omicron variant (BA.4/BA.5) with serum samples from Visits 2 (baseline) and 4 (2 weeks after the booster dose). Blue and red symbols show individual titer values, while bars represent GMTs and error bars represent 95% confidence intervals. Dotted lines indicate the starting dilution (20; lower dotted line) and the final dilution (2560; upper dotted line) for the assay, and all values below 20 are tabulated as 10. Statistical significance was calculated using the Kruskal–Wallis test with a corrected Dunn’s multiple comparisons test.** = *p* < 0.01.

The BA.4/BA.5 pseudovirus neutralization assay was used to investigate immunogenicity against the Omicron variants at the time. In both groups at V4, all types of booster doses had uniformly high levels of neutralizing antibodies against the WT pseudovirus (Figure 4). Against the BA.4/BA.5 pseudovirus, 25 mcg of MVC-COV1901-Beta elicited a significantly higher (*p* < 0.01) level of neutralizing antibodies (ID_50_ 425.7 [272.8–664.2]) than 15 mcg of MVC-COV1901 (ID_50_ 139.1 [76.2–254.1]) (Figure 4, Table S4). In Group B, this was not observed, and instead, all types of booster doses resulted in similar levels of neutralizing antibodies.

**Figure 5:**
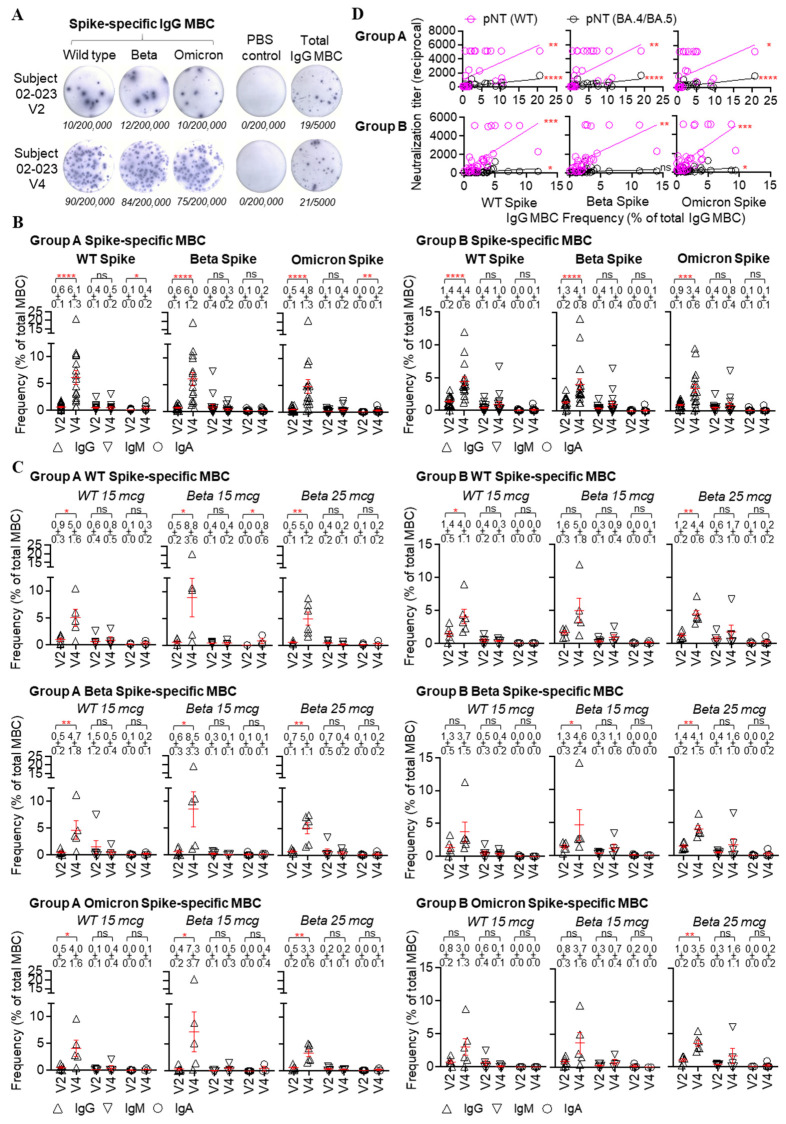
SARS-CoV-2 spike-specific memory B cell (MBC) response before and after the booster dose. (**A**) Illustration of SARS-CoV-2 spike-specific MBC frequency, measured by ELISpot. In the ELISpot assay, antigen-coated wells were used to assess antigen-specific MBC, PBS-coated wells were used as negative controls, and anti-Ig-coated wells were used to assess total IgG MBC. The numbers (in italics) of spots and cultured cells incubated in the ELISpot assay were shown below each image. Each spot represents an antibody-secreting cell. The frequency of antigen-specific IgG MBC was calculated as the percentage of total IgG MBC. Subject 02-023 is an adult who had two prior doses of MVC-COV1901 and received a booster dose of MVC-COV1901 containing a Beta variant spike of 15 mcg. V2, the vaccination day; V4, 14 days after the booster dose. (**B**) spike-specific MBC frequency in the peripheral blood was measured in those with two (group A) and three (group B) initial doses of MVC-COV1901, before (V2) and 14 days (V4) after the booster dose, with the memory B cell ELISpot assay. Wild type (WT), Beta, and Omicron BA.1 spike-specific IgG, IgM, or IgA MBC frequencies were shown in mean ± SEM in the figure. Each symbol represents a sample (subject). (**C**) spike-specific MBC frequency in the subgroups, i.e., booster dose with MVC-COV1901 containing Wuhan wild type spike, booster dose with MVC-COV1901 containing Beta variant spike 15 mcg, and booster dose with MVC-COV1901 containing Beta variant spike 25 mcg, of groups A and B. Wild-type (WT), Beta, and Omicron BA.1 spike-specific IgG, IgM, or IgA MBC frequencies were shown in mean ± SEM in the figure. Each symbol represents a sample (subject). (**D**) Relationship of spike-specific IgG MBC frequency and serological neutralization titer with wild type and Omicron variant BA.4/BA.5 SARS-CoV-2 pseudovirus among group A and B subjects. Linear regression was used to model the relationship between two variables. pNT, pseudovirus neutralization titer. A Mann–Whitney test was used to compare MBC frequencies at two-time points. * *p* < 0.05, ** *p* < 0.01, *** *p* < 0.001, **** *p* < 0.0001. ns, not significant.

Prior to the booster dose at V2, 15 of 18 (83%) Group A subjects had detectable SARS-CoV-2 spike-specific IgG memory B cells (MBCs), in which WT, Beta, or Omicron BA.1 spike-specific IgG cells accounted for around 0.5 to 0.6% of total IgG cells in the peripheral blood (Figure 5A,B; Figure S1). Group B subjects had significantly higher pre-existing WT, Beta, and Omicron BA.1 spike-specific IgG MBC frequencies compared to those of Group A subjects (WT, 0.6 ± 0.1 vs. 1.4 ± 0.2, *p* = 0.002; Beta, 0.6 ± 0.1 vs. 1.3 ± 0.2, *p* = 0.005; Omicron, 0.5 ± 0.1 vs. 0.9 ± 0.1, *p* = 0.029, Mann–Whitney test). Pre-existing spike-specific IgM and IgA MBCs were detected in both groups as well, but there is no significant difference in the frequency between the two groups (Figure 5B). After the booster dose, a significant increase in spike-specific MBC frequency was observed in both Group A and B subjects, of which the IgG MBC response dominated (Figure 5B), followed by the IgM or IgA MBC responses, indicating the elicitation of immune memory to the SARS-CoV-2 spike. At two weeks after the booster dose (V4), the WT, Beta, and Omicron BA.1 spike-specific IgG MBC frequency averaged 6.1 ± 1.3, 6.0 ± 1.2, and 4.8 ± 1.3 of total IgG cells (*p* < 0.0001 for all comparisons between pre-existing and elicited responses), respectively, in Group A (Figure 5B). Enhanced spike-specific IgG MBC responses imparted by the booster were also observed in Group B at day 14 (Figure 5B). Although Group B subjects produced a relatively lower V4 WT, Beta, or Omicron BA.1 spike-specific IgG MBC frequency compared to that of Group A, the difference was not statistically significant.

All treatment groups in Group A elicited a significantly higher WT, Beta, or Omicron BA.1 spike-specific IgG MBC frequency than that at the baseline (Figure 5C). Those with a Beta 15 mcg booster produced a relatively higher wild type, Beta or Omicron BA.1 spike-specific IgG MBC frequency at V4 than those with wild-type or Beta 25 mcg boosters, but the difference was not statistically significant (Figure S2). Within Group B, those with the original MVC-COV1901 booster produced an elevated spike-specific IgG MBC frequency at V4, but did not result in significant increases in Beta or Omicron BA.1 spike-specific MBC frequency (Figure 5C). Those with Beta 15 mcg booster produced a significantly higher Beta spike-specific IgG MBC response at V4, and those with Beta 25 mcg booster produced significantly higher wild-type, Beta, and Omicron BA.1 spike-specific IgG MBC frequencies at V4 are higher than those at the baseline (Figure 5C). Nevertheless, similar WT, Beta, or Omicron BA.1 spike-specific IgG MBC frequencies were detected among three subgroups at V4 (Figure S2).

Significant correlations between spike-specific MBC frequency and serological neutralization titer were observed for Group A and B subjects, indicating a potential role of spike-specific B cell response in the development of antibody immunity upon SARS-CoV-2 immunization (Figure 5D; Figure S3). 

The T cell immune response was investigated by the production of interferon- gamma (IFN-γ) and IL-4 for Th-1 and Th-2 responses, respectively. Results indicated a generally Th-1-biased T cell response based on a higher amount of IFN-γ induction than IL-4 induction, particularly in Group A (Figure S4). 

## 4. Discussion

This trial investigated the safety and immunogenicity of a Beta variant version of the CpG 1018-adjuvanted subunit SARS-CoV-2 vaccine, MVC-COV1901-Beta, for its use as a heterologous booster dose following two or three doses of MVC-COV1901. The safety profile of MVC-COV1901-Beta was in line with that of the original MVC-COV1901, with pain/tenderness and malaise/fatigue as the most common adverse events, while incidences of fever were rarely reported (Figure 2) [14,15].

In this study, boosting with MVC-COV1901-Beta with 25 mcg of Beta S-2P protein has been shown to significantly increase the neutralizing antibody titer against the Beta and BA.1 Omicron variants as well as the BA.4/BA.5 pseudovirus compared to boosting with 15 mcg of MVC-COV1901 following two doses of MVC-COV1901 (Figure 3 and Figure 4). The finding is consistent with our previous study, though, with the prototype antigen, which showed an increase in cross-reactivity against variants when the antigen amount increased while the adjuvant remained unchanged [16]. However, the levels of increase in neutralizing antibody titer were lower in participants who had received three doses of MVC-COV1901 prior to boosting (Group B) (Figure 3 and Figure 4). We attribute this to the differences in intervals between the last dose of MVC-COV1901 and the booster dose, which in Group A ranged from a mean of 223.3 to 294.5 days, while it ranged from a mean of 120.9 to 128 days for Group B (Table 1). As less time has passed in Group B following the last vaccination compared to Group A, the baseline titers for Group B were higher, and the boosting effect was less dramatic than that of Group A. In addition, in the results from our previous study in the course of three doses of MVC-COV1901 vaccination, the rate of neutralizing antibody titer decay was slower after the third (booster) dose compared to the second dose, which could also explain the higher baseline titers in Group B [11]. The increased spectrum of neutralization against variants was also observed previously in our hamster study, in which two doses of MVC-COV1901 followed by a dose of MVC-CV1901-Beta resulted in the highest neutralizing antibody titers against all variants tested compared to other dosing regimens [11].

While homologous booster after two doses of vaccination provided higher protection against hospitalization against the Omicron variant, the vaccine efficiencies against infection still remained poor [17,18]. Moderna has developed a Beta version of its mRNA vaccine, mRNA-1273.351, as well as a bivalent vaccine (mRNA-1273.211) consisting of a mixture of the ancestral and Beta variants as booster doses [19]. Both Beta-based mRNA vaccine candidates showed enhanced neutralization against VOCs of the time (Beta, Gamma, and Delta) when given as boosters following the primary series of mRNA1273; however, the Beta vaccine candidate was not submitted for approval, and Moderna went on and released an Omicron-based bivalent vaccine instead [19]. The newer generation of bivalent mRNA-1273.214 based on the ancestral and Omicron strains imparted a 5.4-fold increase in neutralizing antibody response against the BA.4/BA.5 subvariants in a phase 2/3 clinical trial in participants with three prior doses of mRNA-1273 [20]. Another monovalent adjuvanted subunit vaccine by Sanofi/GSK also generated higher titers of neutralizing antibodies against the Omicron BA.1 variant compared to other vaccines tested when used as a third-dose booster [21]. The Sanofi Beta variant vaccine (VidPrevtyn Beta) was approved by the EU as a booster in November 2022 and remains the only COVID-19 booster vaccine using the Beta variant [22]. The researchers for VidPrevtyn Beta also argued for the use of a Beta variant-based vaccine even during the current Omicron landscape of COVID-19, citing the evidence of broad neutralization against Omicron subvariants and durability of immunity conferred by VidPrevtyn Beta in non-human primate and clinical studies [23,24]. AZD2816, a Beta version of AstraZeneca AZD1222, demonstrated improved neutralization against the Beta variant when AZD2816 was given as a booster dose after AZD1222 or mRNA primary series [25]. However, with the rise of Omicron variants, AZD2816 was dropped from the pipeline by AstraZeneca as the company refocused its strategy [26]. Based on our own data generated in this study and data from the above Beta vaccine candidates, the Beta vaccine remains a viable option for boosters given its ability to induce cross-neutralization even against Omicron variants.

The study by Khoury et al. using the original ancestral strain demonstrated a correlation between the neutralizing antibody levels and the protection from infection [27]. However, given the large variety of Omicron lineage descendants, the correlation between the neutralizing antibody level and protection from the Omicron variant infection has yet to be determined. The revised target product profile for the COVID-19 vaccine published by the WHO in April 2022 reflected the paradigm shift and emphasized the role of a booster vaccine for protection against severe outcomes, including hospitalization and long-term COVID [28]. The T-cell immune response against SARS-CoV-2 is known to play a crucial role in improving the breadth of coverage against variants and offering protection against severe outcomes [29]. In patients with immune-mediated inflammatory diseases on B cell-depleting therapies, breakthrough infections were frequent and associated with severe outcomes [30]. These findings indicate that to achieve the revised target product profile of the COVID vaccine under the era of the Omicron variants, the roles of T and B cell immune responses should be examined as a whole.

The cellular immunity after the booster dose was shown by the proliferation and expansion of spike-specific MBCs, indicating immune memory recall induced by the booster dose (Figure 5). Regardless of the type of booster used, higher proportions of MBC with IgGs specific to WT, Beta, or Omicron spikes were seen after the booster dose, thus MBCs recalled by the booster dose are cross-reactive against all strains tested (Figure 5B,C). The dominance of IgG MBC expansion also reflected the involvement of germinal center reactions and the establishment of spike-specific B cell pools, similar to the findings after the mRNA-1273 boost [31]. These results are also in line with our observation in hamsters in which two doses of WT S-2P plus a dose of Beta S-2P enhanced immunogenicity against the Omicron variant, possibly due to the selection of antibodies targeting the N-terminal, S2, or other conserved residues instead of the immunodominant but highly variable receptor-binding residues [11]. Another study has shown that three homologous doses of vaccination could enhance antigen presentation and expand memory B cells, which can target non-dominant epitopes that are more conserved across different variants [32]. The potency and epitope recognition of spike-specific B cell repertoire elicited by subunit vaccine boost, especially those cross-reacting with the Omicron variant, require further investigation.

One of the limitations of this study includes, as stated above, the differences in the time interval between the last and booster doses in Groups A and B; thus, the two groups could not be compared directly. In the cellular immunity assay, we did not perform surface staining and subpopulation gating, and thus we could not distinguish between single-variant and dual/multi-variant spike-specific MBCs, as has been shown for mRNA-1273 [33]. As MVC-COV1901 is currently only administered in Taiwan, the demographic diversity of subjects is limited. However, in our phase III study in Paraguay with a more diverse set of subjects, we have observed a comparable safety profile to that of phase I and phase II studies conducted in Taiwan [10,15,34]. The small sample size and short duration of follow-up for this study were also not sufficient to compare all the endpoints across treatment arms in terms of immune persistence and efficacy. Moreover, as BA.4/BA.5 variants were circulating during this study, we did not perform a neutralization assay against more recent variants such as XBB, BQ.1, and EG.5.

## 5. Conclusions

In this study, we found that a booster dose with the MVC-COV1901-Beta vaccine after a primary series of MVC-COV1901 can generate a broad immune response that cross-reacts with various Omicron subvariants.

## Figures and Tables

**Table 1:** Demographics and baseline characteristics of the participants for Group A and Group B.

	**Group A (*n* = 45)**			
	**MVC-COV1901** **(15 mcg)**	**MVC-COV1901** **(15 mcg, beta)**	**MVC-COV1901** **(25 mcg, beta)**	*p*-Value *
Number of participants, *n*	15	15	15	-
Age				-
Mean (SD)	36.9 (9.25)	34.7 (7.71)	39.5 (9.16)	0.3309 ^a^
Sex				0.6376 ^b^
Male	8 (53.3%)	11 (73.3%)	9 (60.0%)	-
Female	7 (46.7%)	4 (26.7%)	6 (40.0%)	-
Ethnicity				-
Asian	15 (100%)	15 (100%)	15 (100%)	-
BMI				
Mean (SD)	24.99 (4.14)	24.71 (4.09)	26.21 (5.58)	0.6454 ^a^
Comorbidities				
HIV-positive	0	0	0	-
HBsAg-positive	0	0	0	-
Anti-HCV antibody-positive	0	0	0	-
Cardiovascular disease	0	0	0	-
Cerebrovascular disease	0	0	0	-
Malignancy	0	0	0	-
HbA1c higher than the normal range (%)	0	1 (6.7%)	0	-
1, 2 dose interval (days) mean (SD)	33.1 (6.55)	34.5 (4.21)	32.6 (4.98)	0.3027 ^c^
2, 3 dose interval (days) mean (SD)	-	-	-	0.1595 ^c^
Last dose interval (days) mean (SD)	270.6 (101.06)	223.3 (36.98)	294.5 (90.88)	
	**Group B (*n* = 62)**			
	**MVC-COV1901** **(15 mcg)**	**MVC-COV1901** **(15 mcg, beta)**	**MVC-COV1901** **(25 mcg, beta)**	
Number of participants, *n*	21	21	20	-
Age				-
Mean (SD)	36.8 (9.24)	38.6 (7.53)	38.0 (8.98)	0.8162 ^a^
Sex				0.5530 ^b^
Male	8 (38.1%)	10 (47.6%)	11 (55.0%)	-
Female	13 (61.9%)	11 (52.4%)	9 (45.0%)	-
Ethnicity				-
Asian	21 (100%)	21 (100%)	20 (100%)	-
BMI				
Mean (SD)	24.44 (3.84)	24.21 (4.91)	24.61 (3.97)	0.8172 ^a^
Comorbidities				
HIV-positive	0	0	0	-
HBsAg-positive	0	0	0	-
Anti-HCV antibody-positive	0	0	0	-
Cardiovascular disease	0	1 (4.8%)	0	-
Cerebrovascular disease	1 (4.8%)	0	0	-
Malignancy	0	0	1 (5.0%)	-
HbA1c higher than the normal range (%)	2 (9.5%)	0	1 (5.0%)	-
1, 2 dose interval (days) mean (SD)	38.7 (7.80)	37.5 (3.04)	37.6 (2.78)	0.9774 ^c^
2, 3 dose interval (days) mean (SD)	111.6 (11.82)	113.2 (16.44)	109.2 (9.41)	0.6995 ^c^
Last dose interval (days) mean (SD)	120.9 (12.20)	128.0 (28.05)	123.2 (10.32)	0.7758 ^c^

* *p*-value calculation: a. ANOVA, b. Fisher’s exact test, c. Kruskal–Wallis test.

## Data Availability

The datasets generated during and/or analyzed during the current study are available from the corresponding authors on reasonable request.

## Supplementary Materials

The following supporting information can be downloaded at: https://www.mdpi.com/article/10.3390/vaccines11121798/s1, Figure S1. Summary data of Spike-specific IgG memory B cell frequency prior to (V2) and 14 days after (V4) the booster dose in group A and B subjects; Figure S2. Comparison of Spike-specific IgG MBC frequencies among subgroups in group A and B subjects; Figure S3. Relationship of Spike-specific IgG MBC frequency and serological neutralization titer with authentic wild type and Beta variant viruses among group A and B subjects; Figure S4. Cytokine production induced by MVC-COV1901 or MVC-COV1901-Beta boosters; Table S1. Solicited Local Adverse Events after the Booster Dosing; Table S2. Solicited Systemic Adverse Events after the Booster Dosing; Table S3. Summary of Unsolicited Adverse Events and Other Adverse Events; Table S4. Summary of Immunogenicity Data for Groups A and B.
