# Supplementary material for: Safety, Tolerability, and Immunogenicity of Booster Dose with MVC-COV1901 or MVC-COV1901-Beta SARS-CoV-2 Vaccine in Adults: A Phase I, Prospective, Randomized, Open-Labeled Study
Source: Vaccines (Basel). 2023 Dec 1;11(12):1798. doi: 10.3390/vaccines11121798 (PMC10748207; doi:10.3390/vaccines11121798)
Supplement: Supplementary file 1 [file vaccines-11-01798-s001.zip › vaccines-2700359-supplementary.pdf]

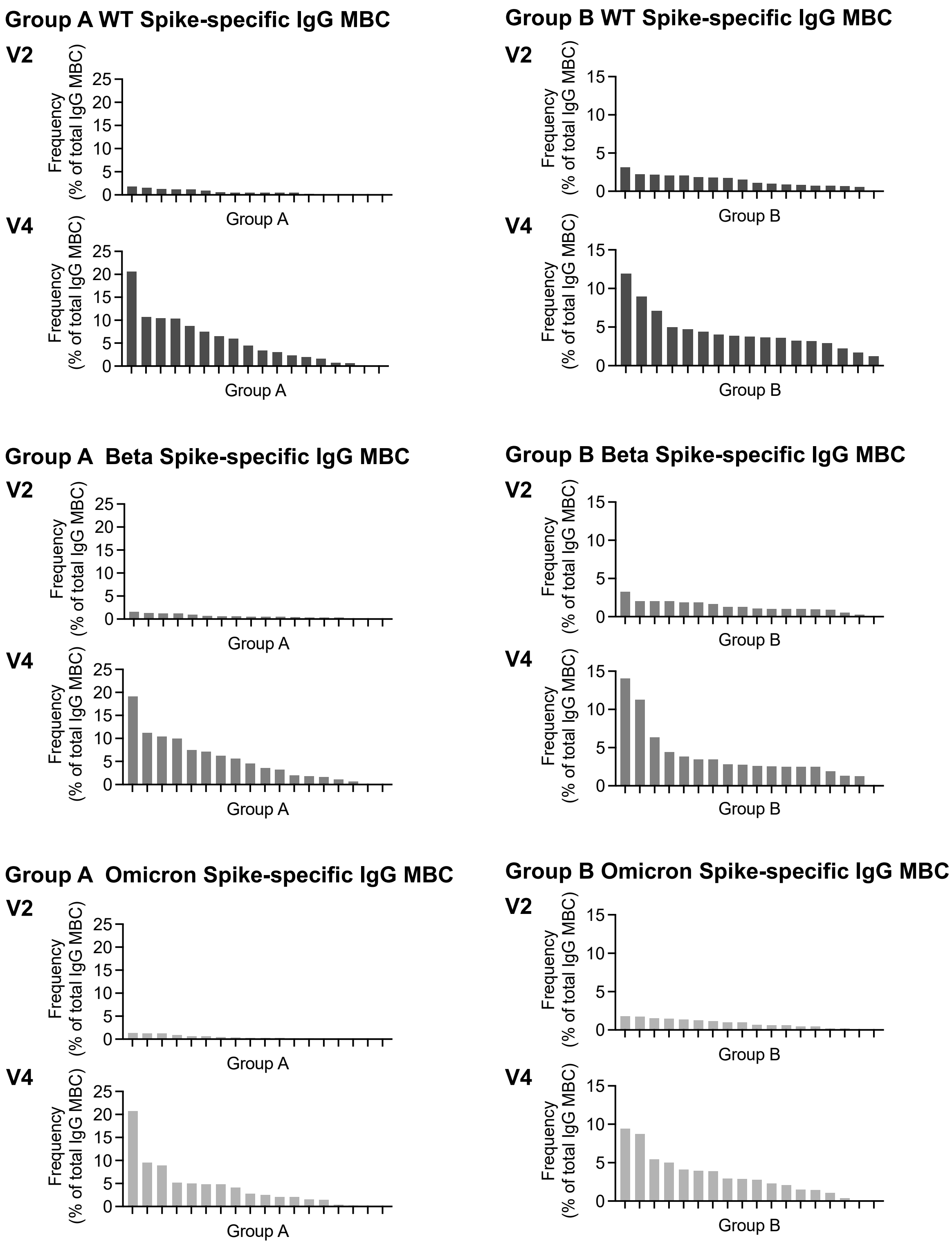

Figure S1. Summary data of Spike-specific IgG memory B cell frequency prior to (V2) and 14 days after (V4) the booster dose in group A and B subjects. Those who had two and three prior MVC-COV191 belonged to group A and B, respectively. WT, wild type. MBC, memory B cell

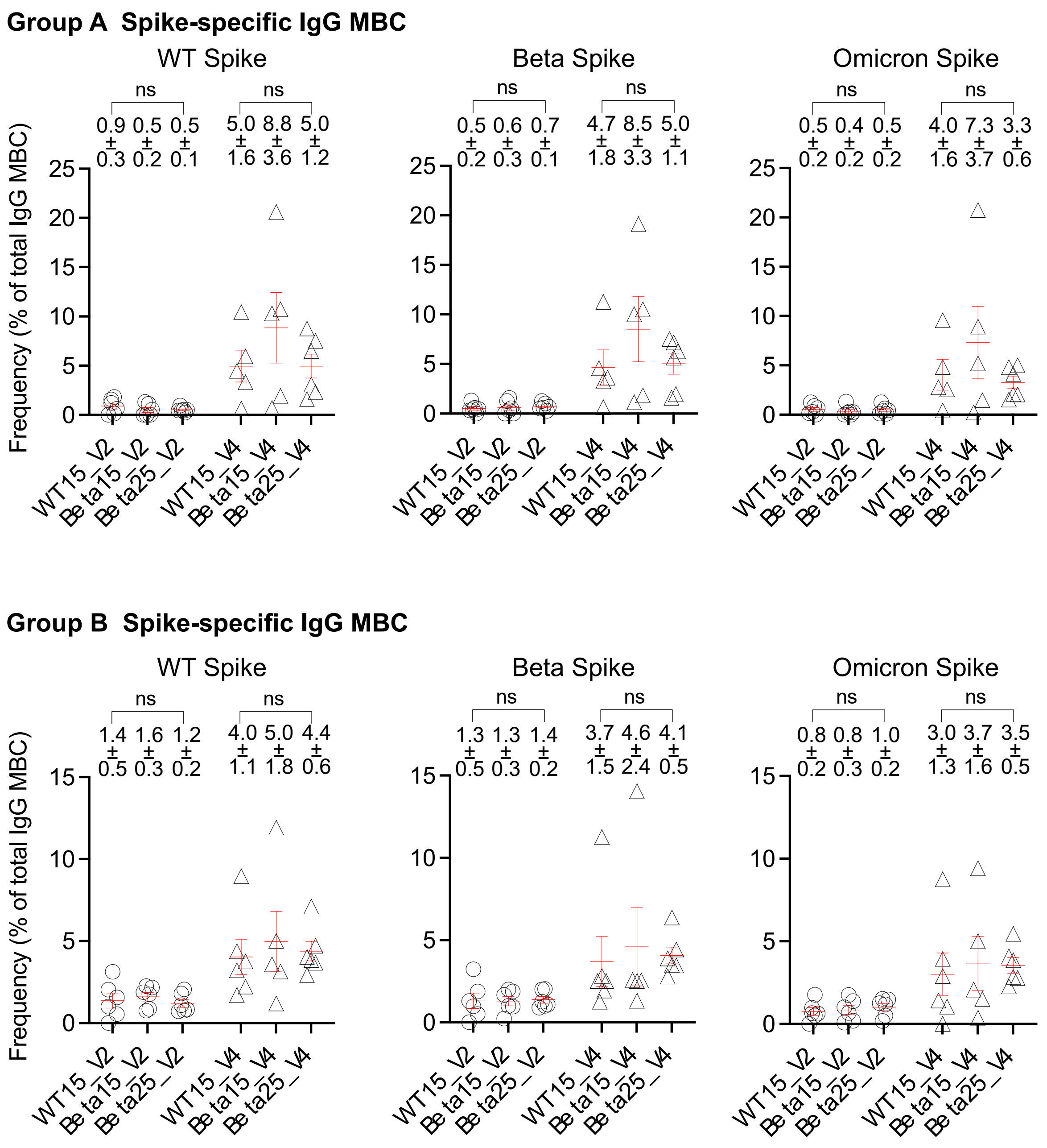

Figure S2. Comparison of Spike-specific IgG MBC frequencies among subgroups in group A and B subjects. There are three subgroups, i.e., booster dose with MVC-COV1901 containing Wuhan wild type Spike, booster dose with MVC-COV1901 containing Beta variant Spike 15 µg, and booster dose with MVC-COV1901 containing Beta variant Spike 25 µg, for each of group A and B. One-way ANOVA was used to compare the difference among subgroups. ns, not significant. V2, the vaccination day; V4, 14 days after the booster dose.

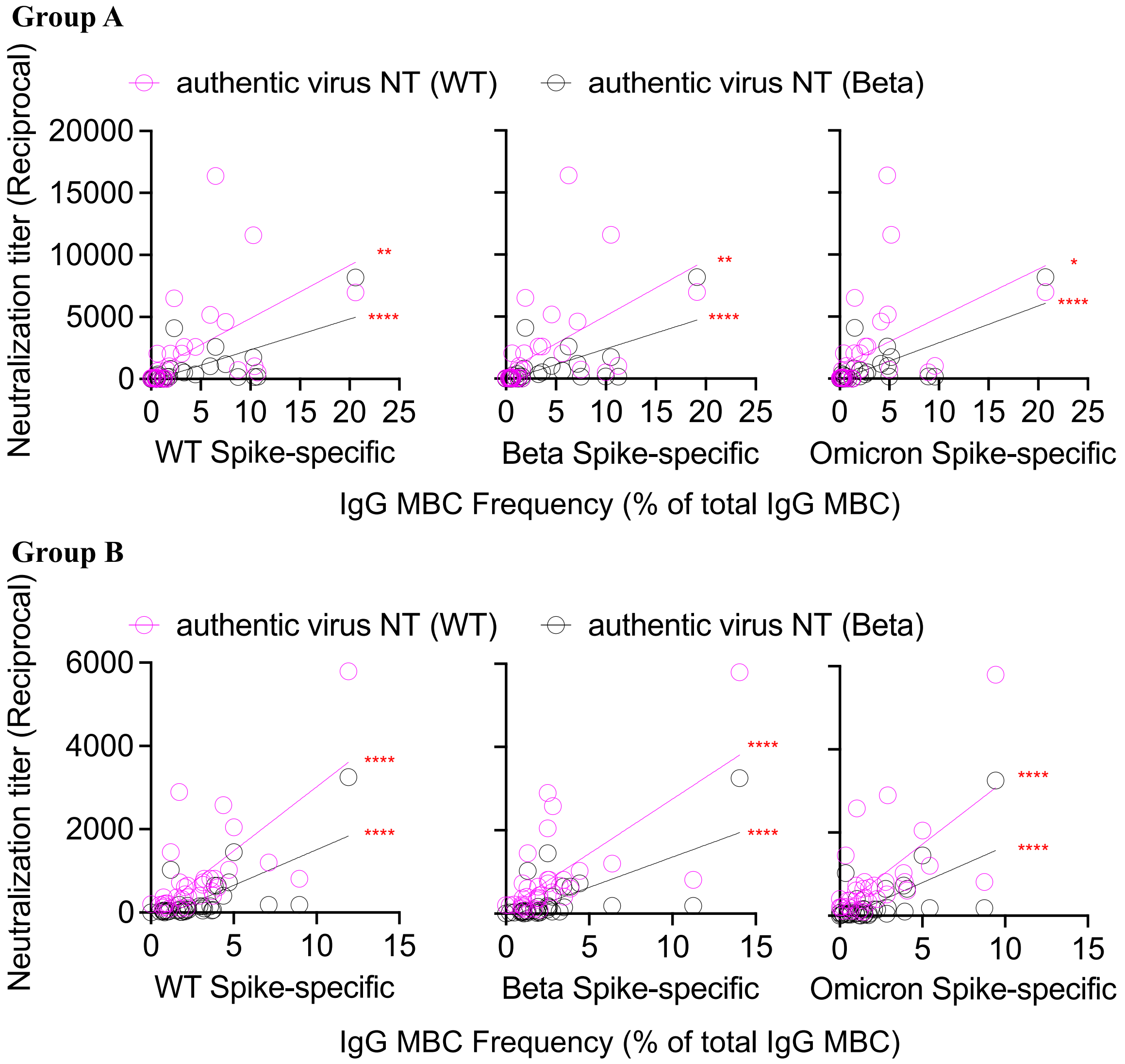

Figure S3. Relationship of Spike-specific IgG MBC frequency and serological neutralization titer with authentic wild type and Beta variant viruses among group A and B subjects. Linear regression was used to model the relationship between two variables. WT, wild type; NT, neutralization test. *p < 0.05, **p < 0.01, ****p < 0.0001.

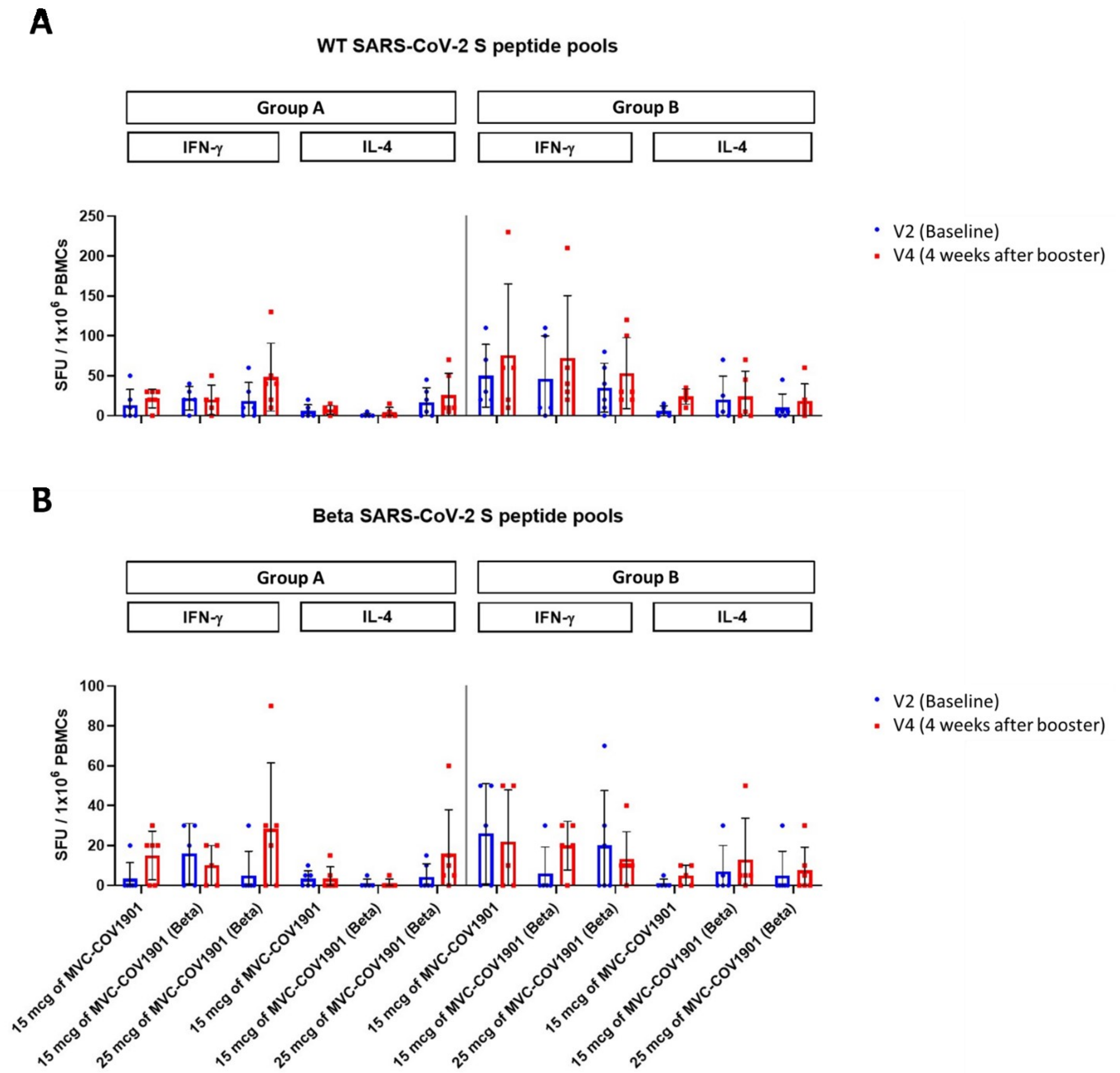

Figure S4. Cytokine production induced by MVC-COV1901 or MVC-COV1901-Beta boosters. PBMC samples were stimulated with peptide pools derived from the ancestral Wuhan (WT) or Beta variant spike protein of SARS-CoV-2. The results are shown in mean spot forming units (SFU) per million of PBMCs with error bars representing standard deviations.

Table S1. Solicited Local Adverse Events after the Booster Dosing

	Group A: Two Prior Doses of MVC-COV1901				Group B: Three Prior Doses of MVC-COV1901			
	**MVC-COV1901 (15 mcg) (N = 15), n (%)**	**MVC-COV1901 (15 mcg, Beta) (N = 15), n (%)**	**MVC-COV1901 (25 mcg, Beta) (N = 15), n (%)**	**All Participants (N = 45), n (%)**	**MVC-COV1901 (15 mcg) (N = 21), n (%)**	**MVC-COV1901 (15 mcg, Beta) (N = 21), n (%)**	**MVC-COV1901 (25 mcg, Beta) (N = 20), n (%)**	**All Participants (N = 62), n (%)**
**Any Solicited Local AEs**	10 (66.7)	11 (73.3)	9 (60.0)	30 (66.7)	13 (61.9)	12 (57.1)	14 (70.0)	39 (62.9)
Grade 1	10 (66.7)	11 (73.3)	8 (53.3)	29 (64.4)	13 (61.9)	11 (52.4)	14 (70.0)	38 (61.3)
Grade 2	0	0	1 (6.7)	1 (2.2)	0	1 (4.8)	0	1 (1.6)
Grade 3	0	0	0	0	0	0	0	0
**Pain/Tenderness**	10 (66.7)	11 (73.3)	9 (60.0)	30 (66.7)	13 (61.9)	12 (57.1)	14 (70.0)	39 (62.9)
Grade 1	10 (66.7)	11 (73.3)	8 (53.3)	29 (64.4)	13 (61.9)	11 (52.4)	14 (70.0)	38 (61.3)
Grade 2	0	0	1 (6.7)	1 (2.2)	0	1 (4.8)	0	1 (1.6)
Grade 3	0	0	0	0	0	0	0	0
**Erythema/Redness**	1 (6.7)	0	1 (6.7)	2 (4.4)	0	0	0	0
Grade 1	1 (6.7)	0	1 (6.7)	2 (4.4)	0	0	0	0
Grade 2	0	0	0	0	0	0	0	0
Grade 3	0	0	0	0	0	0	0	0
**Induration/Swelling**	2 (13.3)	0	3 (20.0)	5 (11.1)	0	1 (4.8)	1 (5.0)	2 (3.2)
Grade 1	2 (13.3)	0	3 (20.0)	5 (11.1)	0	0	1 (5.0)	1 (1.6)
Grade 2	0	0	0	0	0	1 (4.8)	0	1 (1.6)
Grade 3	0	0	0	0	0	0	0	0

Abbreviations: N = number of subjects in the population; n = number of subjects in the specific category. % = percentage of subjects with N as the denominator.

Table S2. Solicited Systemic Adverse Events after the Booster Dosing

	Group A: Two Prior Doses of MVC-COV1901				Group B: Three Prior Doses of MVC-COV1901			
	MVC-COV1901 (15 mcg) (N = 15), n (%)	MVC-COV1901 (15 mcg, Beta) (N = 15), n (%)	MVC-COV1901 (25 mcg, Beta) (N = 15), n (%)	All Participants (N = 45), n (%)	MVC-COV1901 (15 mcg) (N = 21), n (%)	MVC-COV1901 (15 mcg, Beta) (N = 21), n (%)	MVC-COV1901 (25 mcg, Beta) (N = 20), n (%)	All Participants (N = 62), n (%)
**Any Solicited Systemic AEs**	9 (60.0)	9 (60.0)	9 (60.0)	27 (60.0)	9 (42.9)	7 (33.3)	13 (65.0)	29 (46.8)
Grade 1	7 (46.7)	8 (53.3)	6 (40.0)	21 (46.7)	7 (33.3)	6 (28.6)	12 (60.0)	25 (40.3)
Grade 2	1 (6.7)	1 (6.7)	2 (13.3)	4 (8.9)	2 (9.5)	1 (4.8)	0	3 (4.8)
Grade 3	1 (6.7)	0	1 (6.7)	2 (4.4)	0	0	1 (5.0)	1 (1.6)
**Fever**	1 (6.7)	0	1 (6.7)	2 (4.4)	0	0	1 (5.0)	1 (1.6)
Grade 1	1 (6.7)	0	1 (6.7)	2 (4.4)	0	0	0	0
Grade 2	0	0	0	0	0	0	1 (5.0)	1 (1.6)
Grade 3	0	0	0	0	0	0	0	0
**Malaise/Fatigue**	5 (33.3)	7 (46.7)	8 (53.3)	20 (44.4)	7 (33.3)	6 (28.6)	8 (40.0)	21 (33.9)
Grade 1	3 (20.0)	7 (46.7)	5 (33.3)	15 (33.3)	5 (23.8)	5 (23.8)	7 (35.0)	17 (27.4)
Grade 2	1 (6.7)	0	2 (13.3)	3 (6.7)	2 (9.5)	1 (4.8)	0	3 (4.8)
Grade 3	1 (6.7)	0	1 (6.7)	2 (4.4)	0	0	1 (5.0)	1 (1.6)
**Myalgia**	4 (26.7)	3 (20.0)	5 (33.3)	12 (26.7)	5 (23.8)	1 (4.8)	7 (35.0)	13 (21.0)
Grade 1	3 (20.0)	2 (13.3)	4 (26.7)	9 (20.0)	5 (23.8)	1 (4.8)	7 (35.0)	13 (21.0)
Grade 2	1 (6.7)	1 (6.7)	1 (6.7)	3 (6.7)	0	0	0	0
Grade 3	0	0	0	0	0	0	0	0
**Headache**	5 (33.3)	2 (13.3)	4 (26.7)	11 (24.4)	3 (14.3)	3 (14.3)	4 (20.0)	10 (16.1)
Grade 1	4 (26.7)	2 (13.3)	3 (20.0)	9 (20.0)	3 (14.3)	3 (14.3)	3 (15.0)	9 (14.5)

	Group A: Two Prior Doses of MVC-COV1901				Group B: Three Prior Doses of MVC-COV1901			
	MVC-COV1901 (15 mcg) (N = 15), n (%)	MVC-COV1901 (15 mcg, Beta) (N = 15), n (%)	MVC-COV1901 (25 mcg, Beta) (N = 15), n (%)	All Participants (N = 45), n (%)	MVC-COV1901 (15 mcg) (N = 21), n (%)	MVC-COV1901 (15 mcg, Beta) (N = 21), n (%)	MVC-COV1901 (25 mcg, Beta) (N = 20), n (%)	All Participants (N = 62), n (%)
Grade 2	1 (6.7)	0	1 (6.7)	2 (4.4)	0	0	1 (5.0)	1 (1.6)
Grade 3	0	0	0	0	0	0	0	0
**Nausea/Vomiting**	2 (13.3)	1 (6.7)	2 (13.3)	5 (11.1)	0	0	1 (5.0)	1 (1.6)
Grade 1	1 (6.7)	1 (6.7)	2 (13.3)	4 (8.9)	0	0	1 (5.0)	1 (1.6)
Grade 2	1 (6.7)	0	0	1 (2.2)	0	0	0	0
Grade 3	0	0	0	0	0	0	0	0
**Diarrhea**	5 (33.3)	1 (6.7)	2 (13.3)	8 (17.8)	1 (4.8)	3 (14.3)	5 (25.0)	9 (14.5)
Grade 1	4 (26.7)	1 (6.7)	2 (13.3)	7 (15.6)	1 (4.8)	2 (9.5)	5 (25.0)	8 (12.9)
Grade 2	0	0	0	0	0	1 (4.8)	0	1 (1.6)
Grade 3	1 (6.7)	0	0	1 (2.2)	0	0	0	0

Abbreviations: N = number of subjects in the population; n = number of subjects in the specific category. % = percentage of subjects with N as the denominator.

Table S3. Summary of Unsolicited Adverse Events and Other Adverse Events

	Group A: Two Prior Doses of MVC-COV1901				Group B: Three Prior Doses of MVC-COV1901			
	MVC-COV1901 (15 mcg) (N = 15), n (%)	MVC-COV1901 (15 mcg, Beta) (N = 15), n (%)	MVC-COV1901 (25 mcg, Beta) (N = 15), n (%)	All Participants (N = 45), n (%)	MVC-COV1901 (15 mcg) (N = 21), n (%)	MVC-COV1901 (15 mcg, Beta) (N = 21), n (%)	MVC-COV1901 (25 mcg, Beta) (N = 20), n (%)	All Participants (N = 62), n (%)
Unsolicited AEs	4 (26.7)	6 (40.0)	5 (33.3)	15 (33.3)	6 (28.6)	8 (38.1)	3 (15.0)	17 (27.4)
Related Unsolicited AEs	1 (6.7)	1 (6.7)	2 (13.3)	4 (8.9)	2 (9.5)	2 (9.5)	2 (10.0)	6 (9.7)
Unsolicited AEs ≥ Grade 3	0	0	0	0	0	0	0	0
Related Unsolicited AEs ≥ Grade 3	0	0	0	0	0	0	0	0
AEs ≥ Grade 3	0	0	0	0	0	0	0	0
Related AEs ≥ Grade 3	0	0	0	0	0	0	0	0
SAEs	0	0	0	0	0	0	0	0
Related SAEs	0	0	0	0	0	0	0	0
AESI (including pIMD)	0	0	0	0	0	0	0	0
VAED	0	0	0	0	0	0	0	0
AEs Leading to Intervention Discontinuation	0	0	0	0	0	0	0	0
AEs Leading to Study Withdrawal	0	0	0	0	0	0	0	0
Death	0	0	0	0	0	0	0	0

Abbreviations: N = number of subjects in the population; n = number of subjects with events; AE = Adverse Event; SAE = Serious Adverse Event; AESI = Adverse events of special interest; pIMD = potential immune mediated disorders; VAED = Vaccine-associated enhanced disease.

Table S4. Summary of immunogenicity data for Groups A and B

		Group A					
		**MVC-COV1901 (15 mcg) (n=14)**		**MVC-COV1901-Beta (15 mcg) (n=12)**		**MVC-COV1901-Beta (25 mcg) (n=12)**	
		**[GMT]**	**95% CI**	**[GMT]**	**95% CI**	**[GMT]**	**95% CI**
**WT (NT_{50})**	V2 (Day 1)	**61.28**	(34.48, 108.91)	**50.74**	(27.26, 94.44)	**42.47**	(22.82, 79.03)
	V5 (Day 29)	**1352.00**	(797.38, 2292.40)	**1805.02**	(1023.61, 3182.94)	**3602.75**	(2036.68, 6373.05)
**WT GMT ratio**	V5/V2	**26.29**	(15.51, 44.58)	**35.10**	(19.91, 61.90)	**70.06**	(39.61, 123.94)
**Beta (NT_{50})**	V2 (Day 1)	**9.75**	(5.57, 17.08)	**6.29**	(3.43, 11.52)	**5.99**	(3.27, 10.98)
	V5 (Day 29)	**225.59**	(128.13, 397.17)	**931.34**	(509.33, 1703.00)	**1476.85**	(806.39, 2704.76)
**Beta GMT ratio**	V5/V2	**30.98**	(17.60, 54.55)	**127.92**	(69.96, 233.91)	**202.85**	(110.76, 371.51)
**Omicron BA.1 (NT_{50})**	V2 (Day 1)	**6.14**	(3.21, 11.76)	**4.00**	(4.00, 4.00)	**4.00**	(4.00, 4.00)
	V5 (Day 29)	**116.28**	(55.41, 244.04)	**190.39**	(85.92, 421.88)	**609.68**	(280.73, 1324.06)
**BA.1 GMT ratio**	V5/V2	**18.93**	(7.41, 48.33)	**47.61**	(22.49, 100.69)	**152.47**	(73.41, 316.40)
**Anti-spike IgG (WT)**	V2 (Day 1)	**1384.95**	(876.59, 2188.12)	**1301.00**	(793.82, 2132.21)	**1073.76**	(655.17, 1759.79)
	V4 (Day 15)	**25878.60**	(17387.24, 38516.84)	**32938.41**	(21459.28, 50558.02)	**43208.61**	(28085.34, 66475.38)
	V5 (Day 29)	**25257.76**	(16360.67, 38993.18)	**30672.19**	(19210.70, 48971.84)	**61907.00**	(38676.11, 99091.57)
**IgG GMT ratio****	V4/V2	**20.65**	(13.88, 30.74)	**26.29**	(17.13, 40.35)	**34.48**	(22.41, 53.05)
	V5/V2	**20.16**	(13.06, 31.12)	**24.48**	(15.33, 39.08)	**49.41**	(30.87, 79.08)
**Pseudovirus NT ID_{50} (WT)**	V2 (Day 1)	**45.12**	(98.07, 20.76)	**35.97**	(19.84, 65.21)	**40.62**	(26.33, 62.66)
	V4 (Day 15)	**1520.96**	(1023.93, 2259.24)	**1723.40**	(1227.59, 2419.44)	**2315.39**	(1879.03, 2853.08)
**Pseudovirus NT ID_{50} (BA.4/BA.5)**	V2 (Day 1)	**14.50**	(9.39, 22.38)	**10.00**	(10.00, 10.00)	**10.93**	(8.99, 13.30)
	V4 (Day 15)	**139.11**	(76.16, 254.09)	**240.10**	(137.63, 418.86)	**425.65**	(272.79, 664.17)
**Pseudovirus NT ID_{90} (WT)**	V2 (Day 1)	**18.53**	(10.30, 33.33)	**14.58**	(10.82, 19.63)	**14.05**	(10.02, 19.68)
	V4 (Day 15)	**434.71**	(267.37, 706.78)	**558.18**	(328.24, 949.20)	**888.27**	(551.22, 1431.43)
**Pseudovirus NT ID_{90} (BA.4/BA.5)**	V2 (Day 1)	**11.58**	(9.33, 14.38)	**10.00**	(10.00, 10.00)	**10.00**	(10.00, 10.00)
	V4 (Day 15)	**54.52**	(30.41, 97.75)	**92.95**	(57.80, 149.50)	**157.99**	(105.15, 237.39)

		Group B					
		**MVC-COV1901 (15 mcg) (n=17)**		**MVC-COV1901-Beta (15 mcg) (n=18)**		**MVC-COV1901-Beta (25 mcg) (n=18)**	
		**[GMT]**	**95% CI**	**[GMT]**	**95% CI**	**[GMT]**	**95% CI**
**WT (NT_{50})**	V2 (Day 1)	**292.89**	(220.46, 389.11)	**253.89**	(192.56, 334.76)	**263.29**	(198.18, 349.79)
	V5 (Day 29)	**867.93**	(629.98, 1195.74)	**1124.98**	(824.29, 1535.36)	**928.54**	(680.78, 1266.48)
**WT GMT ratio**	V5/V2	**3.25**	(2.36, 4.47)	**4.21**	(3.08, 5.74)	**3.47**	(2.55, 4.74)
**Beta (NT_{50})**	V2 (Day 1)	**34.02**	(21.76, 53.19)	**25.09**	(16.24, 38.77)	**31.17**	(19.94, 48.74)
	V5 (Day 29)	**147.14**	(102.32, 211.61)	**459.23**	(322.19, 654.56)	**323.78**	(227.68, 460.43)
**Beta GMT ratio**	V5/V2	**5.03**	(3.49, 7.23)	**15.69**	(11.01, 22.36)	**11.06**	(7.78, 15.73)
**Omicron BA.1 (NT_{50})**	V2 (Day 1)	**8.92**	(5.48, 14.51)	**6.77**	(4.34, 10.56)	**8.67**	(5.55, 13.52)
	V5 (Day 29)	**54.9**	(36.50, 82.58)	**124.87**	(69.21, 225.29)	**84.89**	(49.50, 145.58)
**BA.1 GMT ratio**	V5/V2	**6.15**	(3.34, 11.34)	**18.45**	(9.05, 37.56)	**9.8**	(4.99, 19.20)
**Anti-spike IgG (WT)**	V2 (Day 1)	**6712.17**	(5261.78, 8562.35)	**4620.77**	(3645.90, 5856.32)	**5307.28**	(4160.46, 6770.21)
	V4 (Day 15)	**21078.01**	(15675.17, 28343.06)	**28179.38**	(21170.75, 37508.24)	**21638.16**	(16216.34, 28872.72)
	V5 (Day 29)	**19014.66**	(14364.79, 25169.70)	**24953.2**	(19040.94, 32701.23)	**21889.96**	(16782.37, 28552.02)
**IgG GMT ratio****	V4/V2	**3.86**	(2.87, 5.19)	**5.16**	(3.87, 6.87)	**3.96**	(2.97, 5.28)
	V5/V2	**3.48**	(2.63, 4.61)	**4.57**	(3.49, 5.99)	**4.01**	(3.07, 5.23)
**Pseudovirus NT ID_{50} (WT)**	V2 (Day 1)	**611.33**	(400.87, 932.27)	**388.14**	(271.86, 554.15)	**408.18**	(254.72, 654.10)
	V4 (Day 15)	**1831.75**	(1449.69, 2314.49)	**1919.17**	(1595.53, 2308.44)	**1919.51**	(1613.19, 2284.00)
**Pseudovirus NT ID_{50} (BA.4/BA.5)**	V2 (Day 1)	**33.28**	(23.69, 46.76)	**22.31**	(15.16, 32.83)	**25.66**	(19.46, 33.84)
	V4 (Day 15)	**136.83**	(93.16, 200.95)	**222.44**	(135.10, 366.22)	**154.62**	(109.03, 219.28)
**Pseudovirus NT ID_{90} (WT)**	V2 (Day 1)	**108.97**	(82.54, 143.87)	**81.60**	(60.18, 110.66)	**92.07**	(64.51, 131.40)
	V4 (Day 15)	**428.68**	(308.92, 594.85)	**533.7**	(360.77, 789.53)	**417.94**	(328.33, 532.01)
**Pseudovirus NT ID_{90} (BA.4/BA.5)**	V2 (Day 1)	**11.85**	(9.69, 14.50)	**11.46**	(9.80, 13.42)	**11.35**	(9.82, 13.12)
	V4 (Day 15)	**50.83**	(35.40, 72.98)	**84.52**	(55.02, 129.83)	**63.74**	(46.65, 87.10)
